# Magnetic stratigraphic dating of marine hydrogenetic ferromanganese crusts

**DOI:** 10.1038/s41598-017-17077-8

**Published:** 2017-12-01

**Authors:** Wei Yuan, Huaiyang Zhou, Xixi Zhao, Zhenyu Yang, Qunhui Yang, Benduo Zhu

**Affiliations:** 1 State Key Laboratory of Marine Geology, Tongji University, Shanghai, 200092 China; 2 Department of Earth and Planetary Sciences, University of California, Santa Cruz, CA 95064 USA; 3 College of Resources, Environment and Tourism, Capital Normal University, Beijing, 100048 China; 4 Key Laboratory of Marine Mineral Resources, Ministry of Land and Resources, Guangzhou, 510075 China; 5 Guangzhou Marine Geological Survey, China Geological Surve, Guangzhou, 510075 China

## Abstract

Deep-sea hydrogenetic ferromanganese crusts are both potential polymetallic resources and records of long-term environmental changes. For palaeoceanographic studies, it is important to construct a detailed and reliable chronological framework. Here, we report the results of a detailed magnetostratigraphic and rock magnetic study of four hydrogenetic Fe-Mn crusts from the Pacific Ocean (PO-01), South China Sea (SCS-01, SCS-02) and Indian Ocean (IO-01). Two groups of characteristic remanent magnetization directions were defined with nearly antipodal normal and reversed polarities for samples PO-01, SCS-01 and SCS-02, indicating a primary record of the Earth’s magnetic field. The magnetostratigraphic framework, established via correlation with the Geomagnetic Polarity Time Scale 2012, implies growth rates of 4.82 mm/Ma, 4.95 mm/Ma, 4.48 mm/Ma and 11.28 mm/Ma for samples PO-01, SCS-01, SCS-02 and IO-01, respectively. Rock magnetic measurements revealed that the Fe-Mn crust samples from the Pacific Ocean and Indian Ocean were dominated by low coercivity, non-interacting, single-domain (SD) magnetite particles, whereas the South China Sea samples were dominated by SD/pseudo-single-domain (PSD) particles. Multidomain (MD) magnetite may also be present in all samples.

## Introduction

Hydrogenetic ferromanganese crusts (hereafter termed Fe-Mn crusts) consist predominantly of seawater-derived ferruginous vernadite (δMnO_2_) and X-ray amorphous Fe oxyhydroxides; they accumulate slowly on hard rock substrates that have been swept clean of sediments for millions of years^1–6^. Thus, they reflect the chemical conditions of the seawater from which they formed. Due to their continuous and extremely slow growth rate (typically several millimetres per million years), Fe-Mn crusts provide a record of the regional and global long-term environmental variations based on temporal changes in radiogenic isotope geochemistry (e.g., Pb, Sr, Nd, Os, Hf), the stable isotope composition of metals (e.g., Fe, Zn, Ni, Cd, Cu, Tl, Mo), and trace metal element distributions^6–9^. Fine-scale analyses are clearly important for correlating the geochemistry of crusts with palaeoceanographic events. The growth dates and ages of Fe-Mn crusts have been determined primarily using U-Th-series isotopes (the ^230^Th half-life is 75.4 ka, which can only be used for the outermost 2 mm) and with ^10^Be/^9^Be isotope ratios (the ^10^Be half-life is 1.387 Myr, which can only be used for the depth interval 0–20 mm)^5,10–12^. In addition, Os isotope stratigraphy has been successfully applied to Fe-Mn crusts, which compares the Os isotope ratios of samples with those that define a Cenozoic seawater curve (crusts as old as 70 Ma can be dated)^6,13^. However, these isotopic techniques are limited by the potential changes in growth rates, the half-lives, and by the difficulty in determining sample thickness, all of which could potentially result in large dating errors.

Palaeomagnetism is an alternative method of dating Fe-Mn crusts, which can provide a high-resolution time framework by correlating the polarity reversal pattern retrieved from a sample with a reference geomagnetic time scale (GPTS)^14–16^. Magnetostratigraphy studies have the potential to provide more control points than typical isotopic age dating methods. Crecelius *et al*.^17^ described the first measurements of the natural remanent magnetization (NRM) of Fe-Mn nodules and demonstrated that they preserved a record of geomagnetic polarity reversals. However, possible movements of the nodule during its growth on the seafloor may have contributed to the incomplete reversal sequence recorded. In contrast, displacement of Fe-Mn crusts formed on hard substrates, such as volcanic seamounts, is unlikely.

An available statistical analysis indicates that 84% of Fe-Mn crusts in the world’s major oceans have growth rates of 1–7 mm/My^5^. With such extremely slow growth rates, fine-scale sample slicing (<~1 mm) is needed to resolve, for example, the geomagnetic Olduvai Normal Chron (1.778–1.945 Ma) within the Matuyama reversed chron. However, Fe-Mn crusts generally have a mean porosity of approximately 60% and a specific surface area that averages 325 m^2^/g, which make it difficult to prepare sub-millimetre scale slice samples. For this reason, it has been very difficult to prepare suitable samples for traditional SQUID magnetometer measurements and subsequently establish a geochronological framework for Fe-Mn crusts.

Consequently, researchers have to cut Fe-Mn crust samples into 2–4-mm-thick slices for magnetostratigraphic age framework studies^18–20^. Due to large potential errors, these studies have, until very recently, failed to convincingly demonstrate the usefulness of palaeomagnetism for dating Fe-Mn crusts. The first successful application of fine-scale magnetostratigraphic determinations on Fe-Mn crusts was reported by Oda *et al*.^21^, who used scanning SQUID microscopy. They found that the average growth rate of the Fe-Mn crusts from the Northwest Pacific Ocean was 5.1 ± 0.2 mm/Ma, which was consistent with the results of ^10^Be/^9^Be isotopic dating. However, for practical reasons this approach cannot be routinely applied in a conventional paleomagnetic laboratory. The latest magnetostratigraphic data indicate that constant ferromanganese crust growth rates of 1.49 mm/Ma–3.67 mm/Ma in the northwest Pacific^22,23^ are consistent with that obtained by ^10^Be/^9^Be geochronology. The results were successfully obtained by using both ordinary SQUID and scanning SQUID microscopy. To remedy the situation and to prepare slices with thicknesses of less than 1.0 mm, we conducted paleomagnetic measurements using a fine saw and a SQUID magnetometer as it is available in a conventional paleomagnetic laboratory.

**Figure 1:**
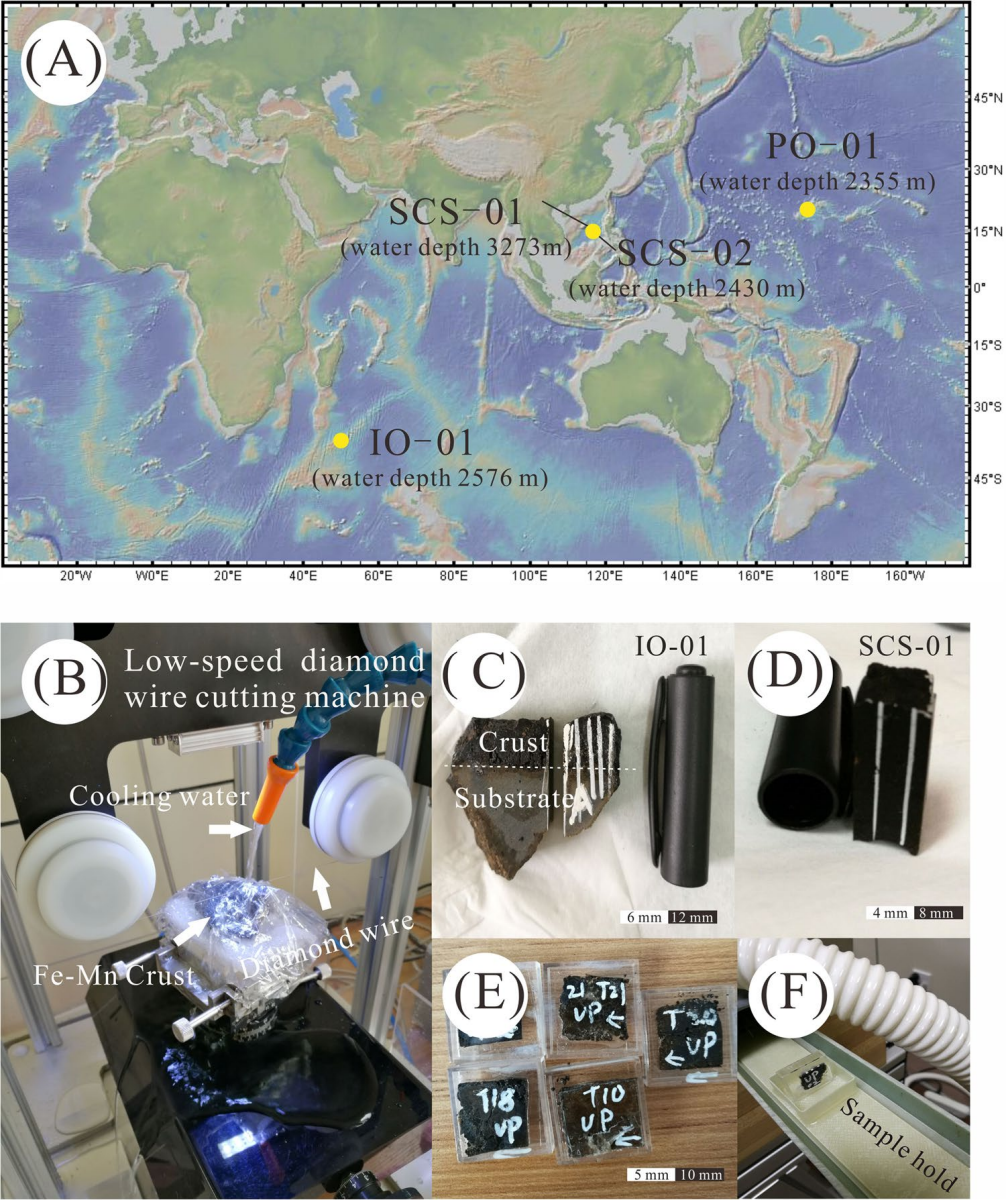
(**A**) Location of the sampling sites in the Pacific (PO-01), South China Sea (SCS-01 and SCS-02) and Southwest Indian Ridge (IO-01). The map was generated with GeoMapApp, http://www.geomapapp.org/); (**B**–**F**) Photographs of the sample and slice.

Fe-Mn crusts from the Pacific have been studied most extensively because they have the greatest economic potential^5,6,17–23^. There are fewer studies of Fe-Mn crusts from the South China Sea and Indian Ocean due in part to the greater input of terrigenous detritus and the topographic dominance of spreading centres. Thus, it is important to investigate the palaeoceanographic conditions in which the Fe-Mn crusts formed in these oceanic areas. During recent research cruises, we obtained Fe-Mn crusts from the Pacific and Indian Oceans and the South China Sea. We have analysed 72 sliced samples from 4 Fe-Mn crusts from the Pacific Ocean, South China Sea and Southwest Indian Ridge (Fig. 1A) using a new type of low-speed diamond wire cutting machine (Model STX-202A, Shenyang Kejing Auto-instrument Co., Ltd.) to obtain sample slice thicknesses of less than 1.0 mm. With samples of this thickness, we could conduct paleomagnetic measurements using a SQUID magnetometer in a conventional paleomagnetic laboratory. In addition to establishing a chronostratigraphic framework, we also determined the composition and grain-size of the magnetic minerals of the hydrogenetic Fe-Mn crusts.

## Samples

Four Fe-Mn crusts were investigated in this study. They were collected from the Pacific Ocean (PO-01, 20°19′N, 174°10′E, water depth of 2355 m), the Indian Ocean (IO-01, 37°47′S, 49°45′E, water depth of 2576 m) and the South China Sea (SCS-01: 15°17′N, 117°34′E, water depth of 3273 m; and SCS-02: 15°09′N, 117°23′E, water depth of 2430 m) with no growth hiatuses (see Fig. 1A). All samples were collected using a trawl net. The Pacific sample was collected during the DY34-II cruise; the Indian Ocean sample was collected during the DY115–18 cruise; and the South China Sea samples were collected on the research vessel ‘Haiyangsihao’ during regional surveys of the South China Sea in 2014. In all cases, the substrate rock and the smooth aspect of the upper surface were used to determine the growth direction (Fig. S1).

Subsamples of Fe-Mn crusts were cut with a new low-speed diamond wire cutting machine (Model STX-202A) (Fig. 1B). The wire diameter is only 0.20 mm, which considerably reduced the amount of material lost during cutting. The thickness lost during cutting needs to be estimated when calculating the growth rate. The slices must be cut perpendicular to the growth axis, and the relative orientation of each slice must be determined. The following sliced samples were obtained: 31 slices with dimensions of 15.5 mm × 14.5 mm × 1.0–1.5 mm from sample PO-01 from the Northwest Pacific, 9 slices with dimensions of 11 mm × 11 mm × 1.0–1.5 mm from sample SCS-01 from the South China Sea, 17 slices with dimensions of 16 mm × 16 mm × 0.4–0.7 mm from sample SCS-02 from the South China Sea, and 15 slices with dimensions of 12 mm × 14 mm × 0.5–0.8 mm from sample IO-01 from the Southwest Indian Ridge. The use of this very fine saw to obtain samples of Fe-Mn crusts with thicknesses of less than 1.0 mm is a major advance of this study and yielded reliable and repeatable paleomagnetic measurements with a SQUID magnetometer in a conventional paleomagnetic laboratory.

## Results

**Figure 2:**
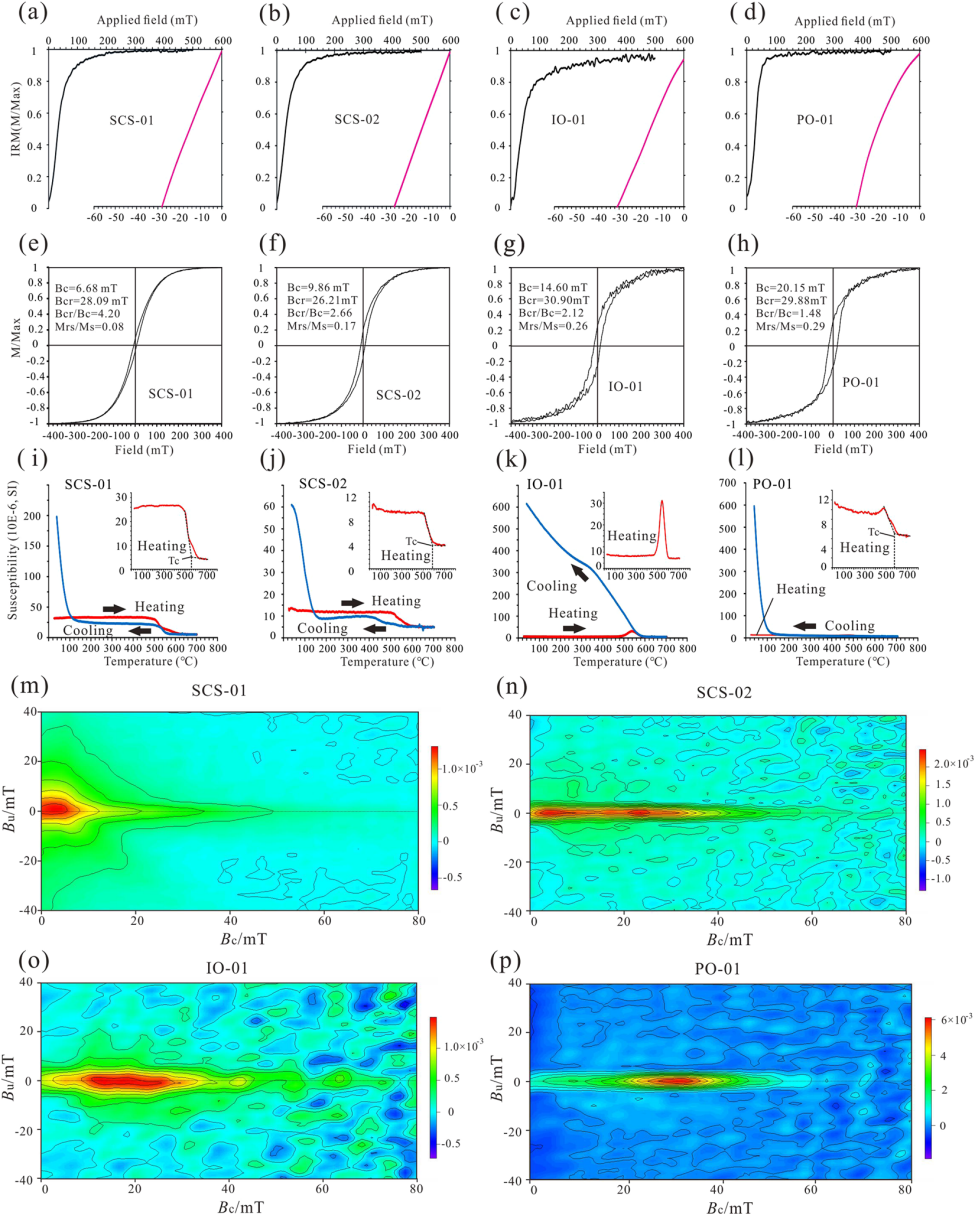
(**a**–**d**) Normalized isothermal remanent magnetization (IRM) acquisition curves (black curves) and back field demagnetization of the IRM (pink curves). (**e**–**h**) Room temperature hysteresis loops. (**i**–**l**) Temperature dependence of magnetic susceptibility (κ–T curves). The red and blue lines indicate heating and cooling curves, respectively.

Isothermal remanent magnetization (IRM) experiments reveal coercivity (Bcr) values within the range of 20–30 mT, which are compatible with a soft, ferromagnetic component such as magnetite (Fig. 2a–d). Variations in the magnetic susceptibility with temperature (κ-T curves) for the four crust samples are shown in Fig. 2i–l. For samples used in this study, both the heating and cooling curves exhibited an obvious Curie temperature of approximately 580 °C indicating magnetite^24–26^ (Fig. 2i,j,l). Sample IO-01 exhibited a significant peak at approximately 540 °C (Fig. 2k), which can be interpreted as the Hopkinson peak of magnetite^27^. The hysteresis loops closed below 400 mT, and the coercivity of remanence (Bcr) was usually less than 30 mT, indicating the presence of low coercivity magnetic minerals (Fig. 2d–f). The FORC diagram is a powerful tool for providing information on the domain state, remanence coercivity, and magnetostatic interaction of magnetic crystals. As shown in Fig. 2(m–p), the FORC diagrams for samples IO-01 and PO-01 were distributed horizontally around coercivity values of 20–30 mT and had rather narrow vertical spreads (Fig. 2o,p). The rock magnetic properties indicated a non-interacting stable SD assemblage of low coercivity magnetic minerals^27,28^. The FORC diagram for SCS-02 was characterized by two independent closed contours, indicating that minerals with two different coercivities coexisted in the sample (Fig. 2n). One peak in the low coercivity had a range of approximately 6 mT, suggesting the presence of superparamagnetic (SP)/SD magnetic particles. The other smoothly closed contours of approximately 20-30 mT indicated the presence of stable SD magnetic particles (Fig. 2n). In contrast to SCS-02, SCS-01 showed much lower coercivity (<5 mT) and a considerably wider vertical spread along the *B*u axis (Fig. 2m). Taken together, these findings suggest that sample SCS-01 shows typical nature of PSD magnetic particles^29^. The magnetic properties suggest that the Fe-Mn crust samples from the Pacific Ocean and Indian Ocean were dominated by low coercivity, non-interacting, single-domain (SD) magnetite particles, whereas the South China Sea samples were dominated by SD/PSD particles. The hysteresis loop parameters Mrs/Ms and Bcr/Bc values ranged from 0.08–0.29 and 1.48–4.20, respectively. These parameters for all the samples were plotted in the pseudo-single-domain (PSD) field in the Day diagram^30,31^ (Fig. S2), suggesting a mixture of SD and multidomain (MD) magnetic minerals in the Fe-Mn crusts.

The results of stepwise AF demagnetization are presented in Table S1. The NRM intensities of the samples varied between 3.86 × 10^−6^ A/m and 5.72 × 10^−3^ A/m. For reference, the intensities of the empty sample holders ranged from 1.0 × 10^−8^ A/m to 1.0 × 10^−7^ A/m. The NRM was almost completely demagnetized in a peak field of 60 mT and typically exhibited two stable remanence directions (Fig. S3a–p). In most cases, a soft component was removed below 20 mT demagnetization and most likely represents a magnetic overprint induced during sampling or a viscous remanent magnetization (Fig. S3(m–n)). Typically, a hard and stable component was also present that decayed linearly towards the origin of the vector plots and was completely removed in a maximum AF demagnetization field of 60–80 mT. We interpret this stable remanence as the characteristic remanent magnetization (ChRM).

## Discussion

The sampling sites in the Pacific Ocean and South China Sea were located at low to middle latitudes in the Northern Hemisphere; therefore, positive, downward-directed inclination values of samples should indicate normal polarity. The topmost slice samples from the Fe-Mn crust commonly yielded coherently positive inclinations, indicating the records of Brunhes normal polarity chron. In contrast, the Southwest Indian Ridge sampling site was in the Southern Hemisphere; therefore, negative, upward-directed inclination values indicate normal polarity. Furthermore, because the substrate rock was probably not lying horizontally, it is necessary to combine the inclinations with the nearly 180 degree change in relative declination values to estimate the magnetic polarity.

For the 31 slice samples of Fe-Mn crust PO-01 from the Northwest Pacific, a secondary magnetization component was removed at 10 mT, and the ChRM was isolated between 12.5 mT and 60 mT. The ChRM of the slice sample from a depth of 0–2.52 mm resulted in an inclination of 61.6° and a relative declination of 219.6° (Table S1, Fig. S3(m)), while the sample from 2.94–3.94 mm resulted in an inclination of −66.8° and a relative declination of 59.8° (Table S1, Fig. S3(n)). ChRM directions exhibited both normal and reversed polarities that are roughly antipodal, suggesting that they are a primary record of the Earth’s magnetic field reversals (Fig. S3d). The mean inclination of the 31 slices was 49.6° (N = 31, a95 = 9.2°), which is slightly higher than the expected magnetic inclination (36.5°) for the site latitude. Considering that the substrate rock was probably not lying horizontally, the mean inclination we obtained is considered acceptable.

**Figure 3:**
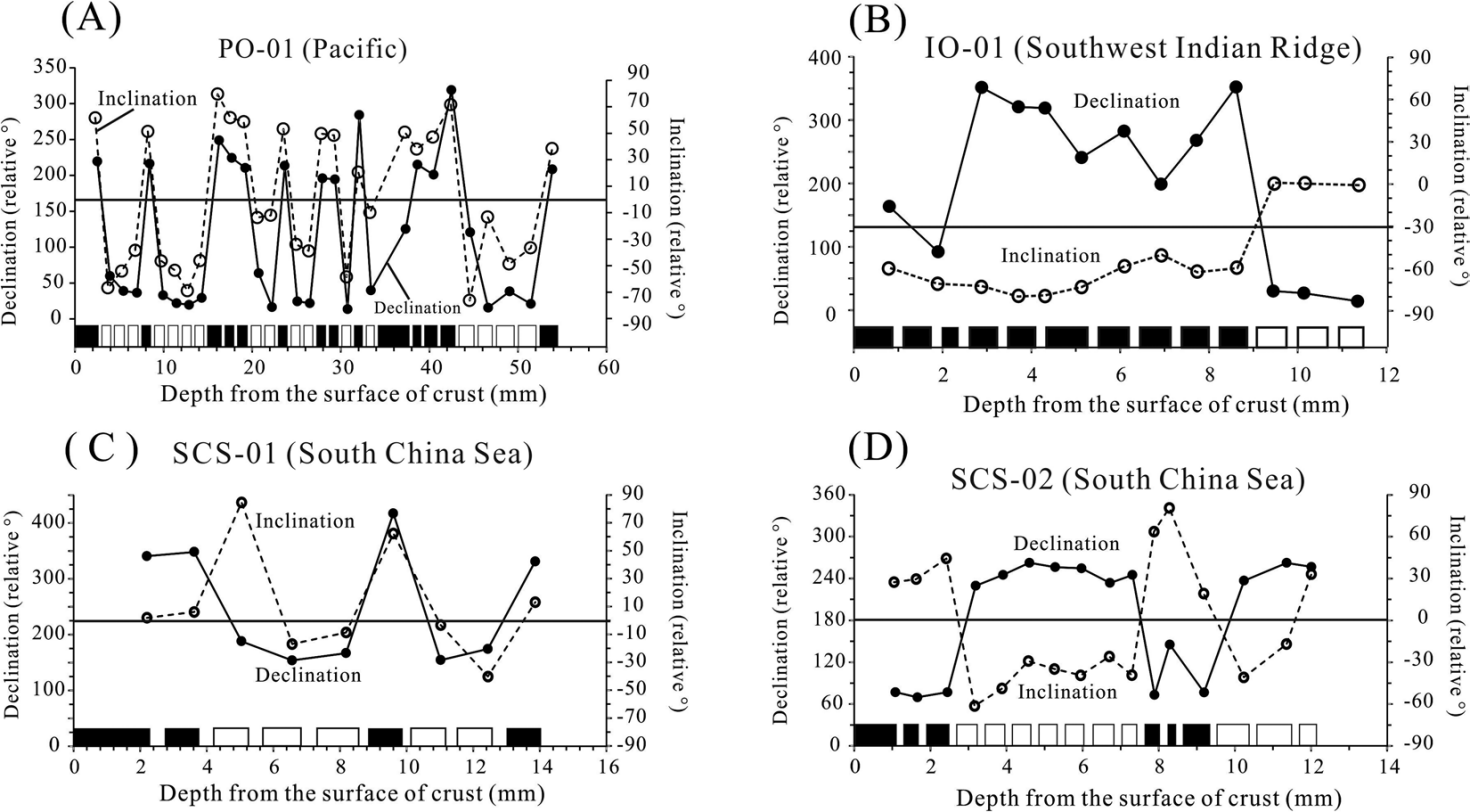
Inclination and declination plotted versus distance of sample from the surface of the crust, and the observed polarity zones. (**A**) Sample PO-01 from the Pacific Ocean; (**B**) sample IO-01 from the Southwest Indian Ridge; (**C**) and (**D**) samples SCS-01 and SCS-02 from the South China Sea. An interpreted polarity for each slice is indicated at the bottom of the diagram: normal polarity is marked in black, reversed polarity in white; the space between the black squares and the white squares represents the loss during cutting. The width of the black squares and white squares represents the thickness of slices corresponding to the x-axis. The full circles represent declination, and open circles represent inclination.

**Figure 4:**
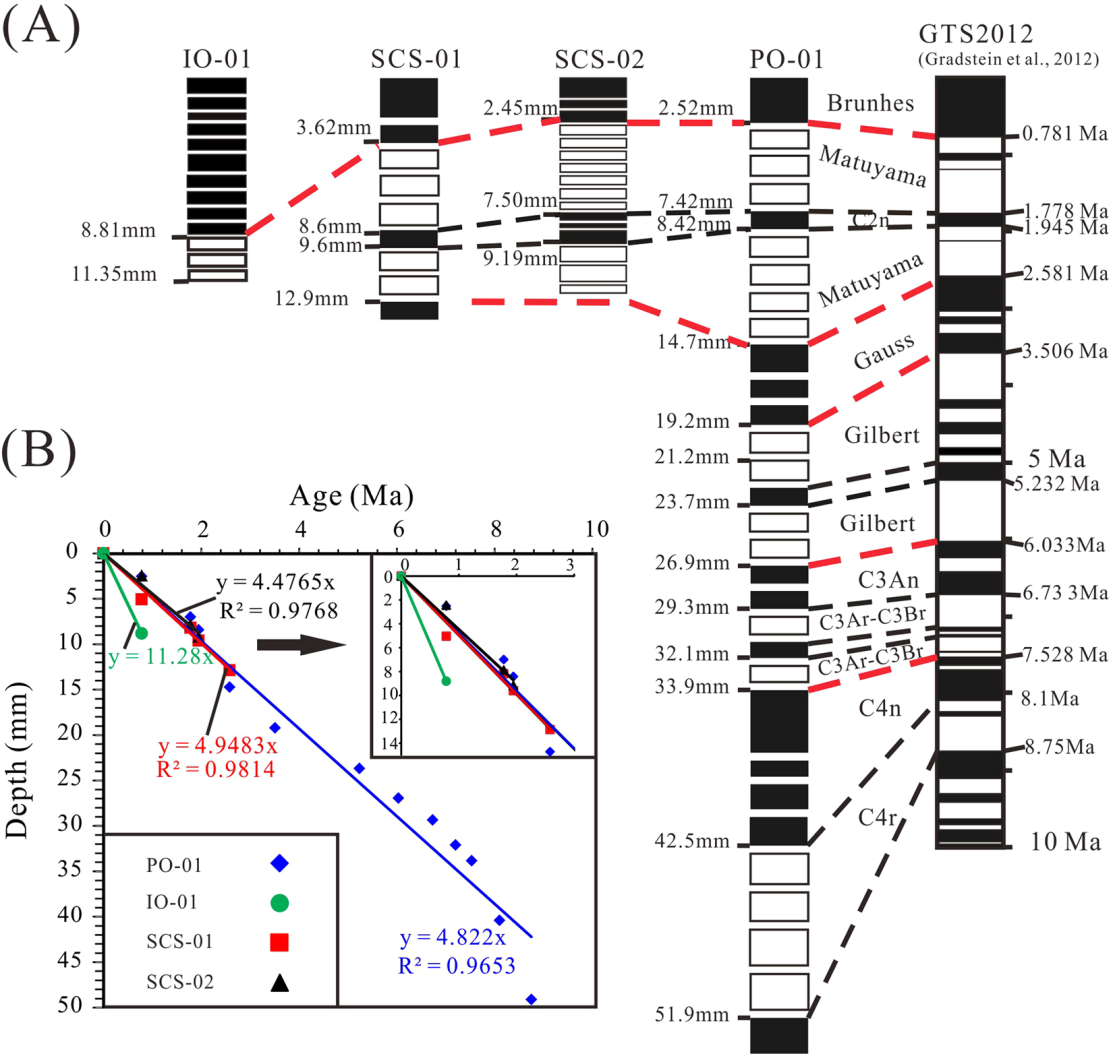
(**A**) Magnetostratigraphic interpretation of the ferromanganese crusts and correlation to GPTS 2012^16^. The red and black dashed lines indicate the correlations of the major chron and subchron boundaries, respectively. (**B**) Depths were plotted versus age based on the magnetostratigraphy.

The slices from Fe-Mn crust PO-01 record 8 normal and 7 reversed polarity zones (Figs 3A and 4A). Comparison with the geomagnetic polarity timescale (GPTS)^16^ suggested the following correlations (Fig. 4A): 0–2.52 mm, Brunhes normal chron (0–0.781 Ma); 2.94–14.3 mm, Matuyama reversed chron (0.781–2.588 Ma); 14.72–19.21 mm, Gauss normal chron (2.588–3.506 Ma); 19.63–26.51 mm, Gilbert reversed chron (3.596–6.033 Ma); 26.93–29.35 mm, chron C3An (6.033–6.733 Ma); 29.77–33.41 mm, chron C3Ar-C3Br (6.733–7.528 Ma); 33.83–42.49 mm, chron C4n (7.528–8.10 Ma); and 51.47 mm, the upper boundary of chron C4r (8.75 Ma). In addition, at a depth interval of 7.42–8.42 mm is the Olduvai normal subchron (1.778–1.945 Ma) within the Matuyama reversed chron (0.781–2.588 Ma). These results indicate a maximum age of 8.75 Ma for the thickest Fe-Mn crust PO-01, from the Northwest Pacific.

Samples SCS-01 and SCS-02 were obtained from the South China Sea. SCS-01 recorded 2 normal and 3 reversed polarity zones (Fig. 3C), and SCS-01 yielded 2 normal and 2 reversed polarity zones (Fig. 3D). The mean inclinations of samples SCS-01 and SCS-02 were 26.6° (N = 9, a95 = 25.8°) and 34.3° (N = 17, a95 = 12.8°), respectively, which are consistent with the expected magnetic inclination (28.5°) for the site latitude. In sample SCS-01, the slices from the depth intervals of 0–2.2 mm and 2.62–3.62 mm had inclination values of 1.9° and 13.8° and declination values of 340.6° and 348.1°, respectively. Thus, they were of normal polarity and should be correlated with the Brunhes normal chron (0–0.781 Ma) (Fig. 4A). The slices from the depth intervals of 5.04–6.56 mm and 6.98–8.18 mm had inclination values of −16.9° and −8.6° and declination values of 153.8° and 166.8°, respectively. They are obviously in the antipodal direction and thus can be considered as a geomagnetic reversal record. The other data values were similar and indicate roughly opposite normal and reversed directions (Table S1, Fig. S3a).

In sample SCS-02, the slices depth intervals of 11.56–12.01 mm (from the bottom of the crust) and 12.21–14.84 mm (from basaltic substrate) had inclinations of 33.2° and 5.7°, respectively (Table S1). However, the respective declinations were 256.6°and 266.4°, similar to those of the reversed polarity specimens (Table S1). The sampling site in the South China Sea was at a low latitude (~15°N), and the substrate rock was probably not in a horizontal position. Therefore, it is necessary to combine the inclinations with the relative declination values to estimate the magnetic polarity. We thus propose that these two slices were of reversed polarity. In sample SCS-01, the slice depth intervals of 4.04–5.04 mm had similar situations (Table S1), which should be defined as the reversed polarity (Fig. 3C).

The interval of normal polarity in the depth intervals of 0–3.62 mm in SCS-01 and 0–2.45 mm in SCS-02 was correlated with the Brunhes normal chron (0–0.781 Ma) (Fig. 4A). Similarly, the short, reversed polarity intervals from 8.6–9.6 mm in sample SCS-01 and from 7.51–9.19 mm in SCS-02 was correlated with the Olduvai normal subchron (1.778–1.945 Ma) in the Matuyama reversed chron (Fig. 4A). The normal polarity interval at the base of sample SCS-01 was correlated with the Gauss normal chron, whereas the reversed polarity interval at the base of sample SCS-02 falls within the Matuyama reversed chron (Fig. 4A).

Sample IO-01 was obtained from the Southwest Indian Ridge, which is in the Southern Hemisphere. Therefore, negative, upward-directed inclination values should indicate normal polarity. The ChRM of the slice sample from a depth of 4.51–5.13 mm yielded an inclination of −73.1° and a relative declination of 240.8° (Table S1, Fig. S3(m)), while that of 9.65–10.14 mm resulted in an inclination of 26.9° and a relative declination of 0.7° (Table S1, Fig. S3(j)). Because the sample was collected using a trawl net from the Southwest Indian Ridge and the substrate rock may not lie in a horizontal position, it is necessary to combine the inclinations with the relative declination values to estimate the magnetic polarity. We defined the southwest and steeply upward inclination as normal polarity and the northeast and shallow, positive inclinations as reversed polarity. The mean inclination of the 15 slices was 57.8° (N = 15, a95 = 25.3°), which is consistent with the expected magnetic inclination (57.2°S) for the site latitude. Two other slice samples IO-01-14 and IO-01-15, which were cut from the basaltic substrate, also exhibited reversed polarity (Table S1).

In this Fe-Mn crust, one normal (0–8.61 mm) and one reversed (8.81–11.35 mm) polarity interval was identified, while the substrate specimen had a reversed polarity (Figs 3B and 4A; Table S1). The long interval of the normal polarity at a depth interval of 0–8.81 mm was correlated to the Brunhes normal chron (0–0.781 Ma), and the reversed polarity at the base of the crust was correlated with the Matuyama chron.

The magnetostratigraphic ages are summarized in Table S1 and Fig. 4A. Based on the magnetostratigraphic ages, the crustal growth rates of samples PO-01, SCS-01, SCS-02 and IO-01 were 4.82 mm/Ma, 4.95 mm/Ma, 4.48 mm/Ma and 11.28 mm/Ma, respectively (Fig. 4B). The growth rate of sample IO-01, with two controlling points (Fig. 4B), was significantly higher than those of the other three samples. Fe-Mn crusts from the Southwest Indian Ridge are always associated with a spreading centre, and therefore, they may have a hydrothermal component, which typically has a growth rate exceeding 12 mm/My^5,6^. Hein and Koschinsky^6^ had analysed the growth rates for hundreds of Fe-Mn crusts from the Indian Ocean, Atlantic Ocean and Pacific Ocean using Be, U, and Os isotopes. These data indicate that more than 75% of the growth rates fall between 1 and 5 mm/Ma^5,6^. These data indicate that the conventional 2G Enterprises cryogenic magnetometer (model 760) can be used to obtain fine-scale magnetostratigraphic ages of Fe-Mn crusts, which can provide a reliable absolute chronological framework. Nevertheless, we still need to consider the possibility of misinterpretation of magnetostratigraphy, e.g., multiple polarities could be included in a single thin section, the uncertainties possibly originated from the flat shape of the samples and potential hiatuses in the growth of Fe-Mn crust.

Hydrogenetic Fe-Mn crusts are usually considered to form by precipitation of the dissolved and detrital components in seawater^32^. The mineral composition consists predominantly of poorly crystalline Fe-bearing vernadite, amorphous iron oxyhydroxides, and minor amounts of detrital minerals such as quartz and feldspar^6^. In this study, the rock magnetic measurements of the Fe-Mn crusts from different oceans yielded similar results. The κ–T heating curves reveal a decrease in magnetic susceptibility at approximately 580 °C, which suggests that Ti-poor magnetite is the major contributor to the susceptibility (Fig. 2i–l). Furthermore, the FORC diagram, hysteresis loops and IRM experiments revealed that the Fe-Mn crust samples from the Pacific Ocean and Indian Ocean were dominated by low coercivity, non-interacting single domain (SD) magnetite particles, whereas the South China Sea samples were dominated by SD/PSD particles.

Fe-Mn crusts have a mean porosity of approximately 60% and an extremely large specific surface area that averages 325 m^2^/g^6^. These properties facilitate surface redox reactions and the adsorption of metals. Paleomagnetic study of ferromanganese crusts from different oceans have shown that the ChRM directions were almost completely antipodal, indicating a primary remanence and that the specimens have reliably recorded magnetic polarity reversals. These data, together with our newly discovered crystalline SD magnetite phase in the hydrogenetic Fe-Mn crusts, lead us to conclude that the primary remanence was acquired during their crystallization in seawater or even during previous weathering processes. That is, the major magnetic contributor, SD magnetite, is a hydrogenetic mineral that formed during the growth of the crust and is not of diagenetic origin, formed within the crust by recrystallization from pseudo-crystallized or amorphous iron oxides or hydroxides^5,6,33,34^.

## Methods

The thickness and weight of each of the slices were determined before measurement (Table S1). Then, the samples were fixed with glue at the centre of the non-magnetic cover of a plastic sample box with dimensions of 20 mm × 20 mm × 5 mm (Fig. 1E). Each sample was positioned in the centre of the magnetometer sample holder (Fig. 1F). To maintain the sample in a constant position and to increase the reliability of the results, the automatic measurement mode was used during the AF demagnetization and remanence measurements. The shape effect was investigated with a basalt cylinder sample and a thin disk sample cut from the cylinder (Fig. S5, Table S2). The results show a directional difference of around 8°, which is considered trivial for the identification of polarity of magnetization.

All the sliced specimens were subjected to stepwise alternating field (AF) demagnetization, and the remanence was measured using a 2G Enterprises cryogenic magnetometer (model 760), which was installed in a magnetically shielded space (<200 nT) at the Paleomagnetic Laboratory of Tongji University, Shanghai, China. To characterize the remanent magnetization of the samples, stepwise AF demagnetization was conducted using 12–15 steps (at 5 mT steps from 0 to 50 mT and at 10 mT steps from 50 to 80 mT), and the results were analysed using principal component analysis^35^. The data analysis was conducted using the paleomagnetic software developed by Enkin^36^ and Cogné^37^.

The Curie temperature is a sensitive indicator of the magnetic mineralogy of a specimen. The κ-T curves for representative samples were obtained from room temperature up to 700 °C and back to room temperature, using a Kappabridge magnetic susceptibility metre (Model MFK1-FA) equipped with a CS-3 high-temperature furnace (AGICO Ltd., Brno, Czech Republic) and operating at a frequency of 976 Hz. To minimize oxidation that could lead to chemical alteration, the measurements were made in an argon atmosphere. The κ-T curves were measured at the Paleomagnetism Laboratory of Tongji University. To estimate the domain status of the magnetic minerals in the hydrogenetic Fe-Mn crusts, hysteresis loops were measured on representative samples. The two ratios, Mrs/Ms and Bcr/Bc, are commonly used as indicators of domain state, and indirectly, the magnetic grain size^30^. Thus, they are useful for assessing the origin of the remanence. Hysteresis curves, isothermal remanent magnetization (IRM) and first-order reversal curves (FORC) (Fig. 2) were measured using a MicroMag 3900 at the Institute of Geology and Geophysics, Chinese Academy of Sciences. FORC diagrams were produced using FORCinel version 1.05 software, with a smoothing factor (SF) of 6^38^. In a FORC diagram, coercive field (*B*c) and magnetostatic interaction fields (*B*u) are indicated on the horizontal and vertical axes, respectively.

## Electronic supplementary material

supplementary material
