# Supplementary material for: Magnetic stratigraphic dating of marine hydrogenetic ferromanganese crusts
Source: Sci Rep. 2017 Dec 1;7:16748. doi: 10.1038/s41598-017-17077-8 (PMC5711896; doi:10.1038/s41598-017-17077-8)
Supplement: Supplementary file 1 — supplementary material [file 41598_2017_17077_MOESM1_ESM.pdf]

Supporting Information for

Magnetic stratigraphic dating of marine hydrogenetic ferromanganese crusts

Wei Yuan[1]* Huaiyang Zhou[1]* Xixi Zhao[1,2] Zhenyu Yang[3] Qunhui Yang[1] Benduo Zhu[4,5]

[1]State Key Laboratory of Marine Geology, Tongji University, Shanghai 200092, China. [2]Department of Earth and Planetary Sciences, University of California, Santa Cruz, CA 95064, USA. [3]College of Resources, Environment and Tourism, Capital Normal University, Beijing 100048, China. [4]Key Laboratory of Marine Mineral Resources, Ministry of Land and Resources, Guangzhou 510075, China. [5]Guangzhou Marine Geological Survey, China Geological Surve, Guangzhou 510075, China.

*These authors contributed equally to this work. Correspondence and requests for materials should be addressed to W.Y. (email: yuanwei@tongji.edu.cn)

Contents of this file

Figures S1- S5
Table S1-S2

Introduction

The supporting information provides the figures and table related to the discussion in the main text.

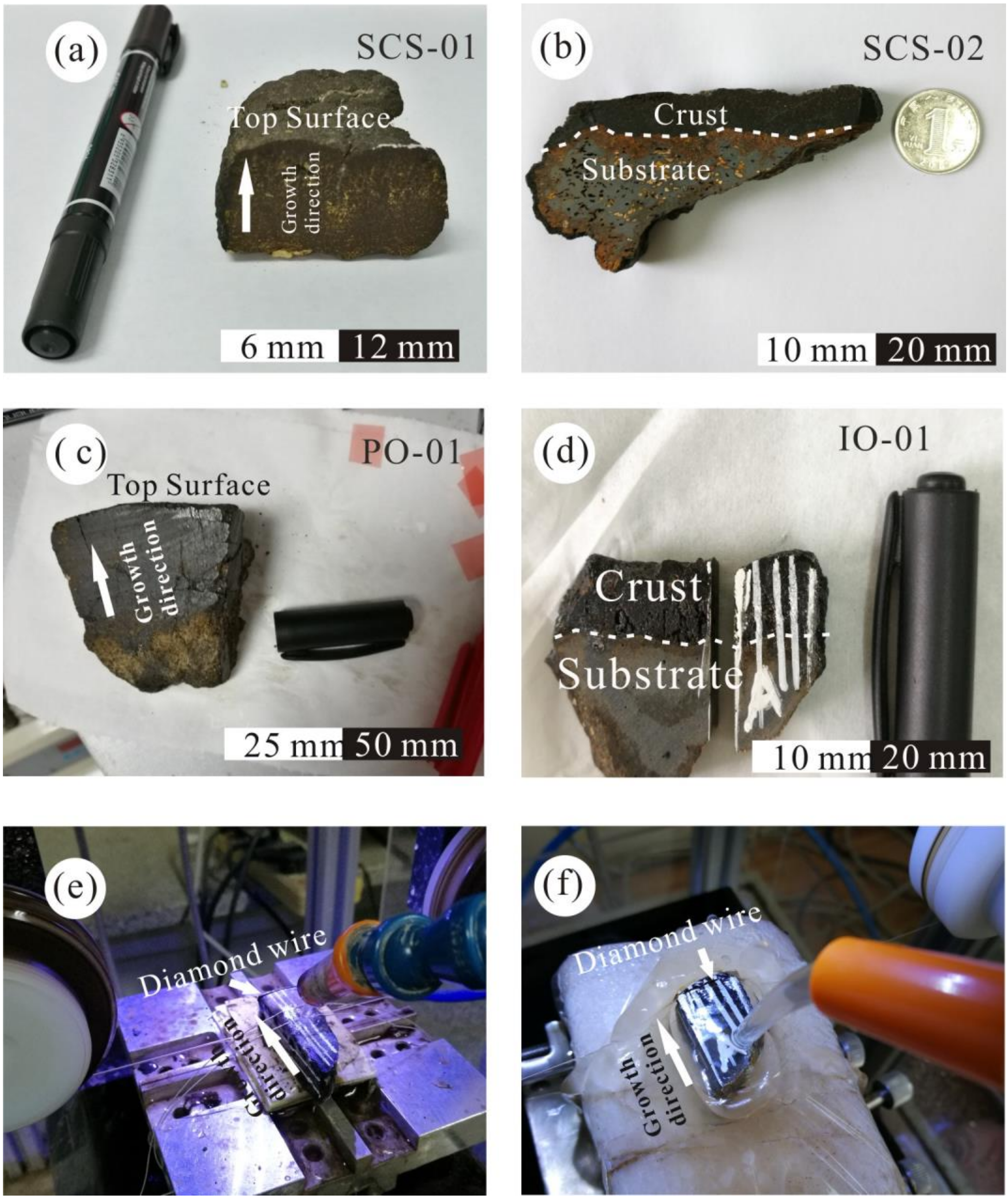

Figure S1. Photographs of the sample and slice preparation procedure.

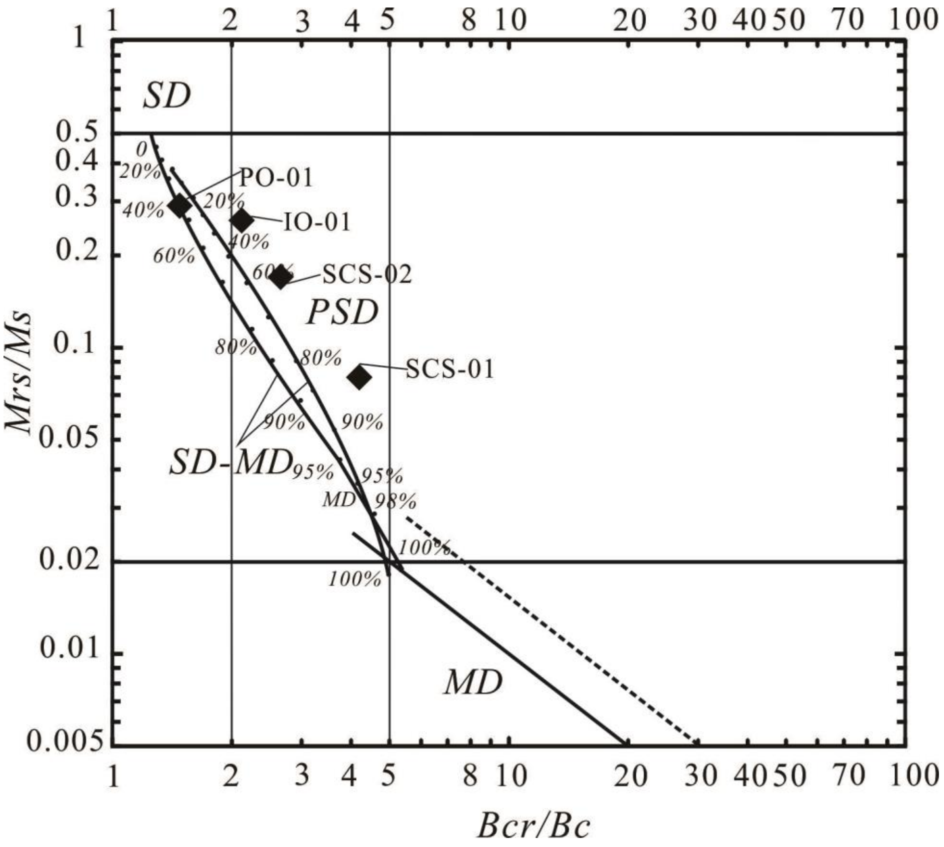

Figure S2. Day plot of the Fe-Mn curst samples for SCS-01, SCS-02, IO-01 and PO-01 (Dunlop, 2002); SD = single domain; MD = multi domain; PSD = pseudo-single domain.

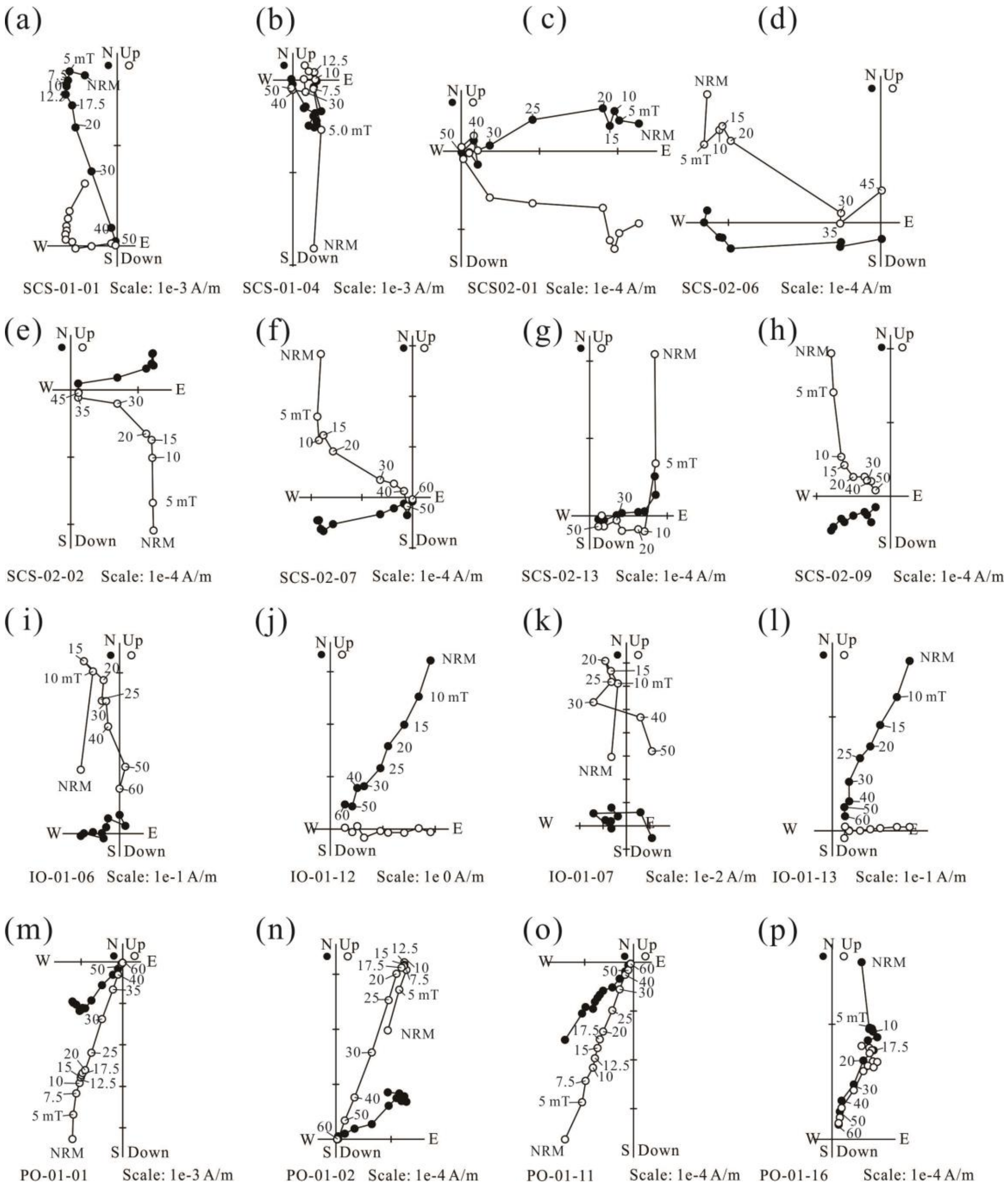

Figure S3. Examples of vector end point diagrams for slices from different sample during AF demagnetization. Samples in (a), (c), (e), (g), (i), (k), (m) and (o) are normally magnetized; and samples in (b), (d), (f), (h), (j), (l), (n) and (p) are reverse magnetized. The solid and open circles represent projection of the magnetization vector end point on the horizontal and vertical planes.

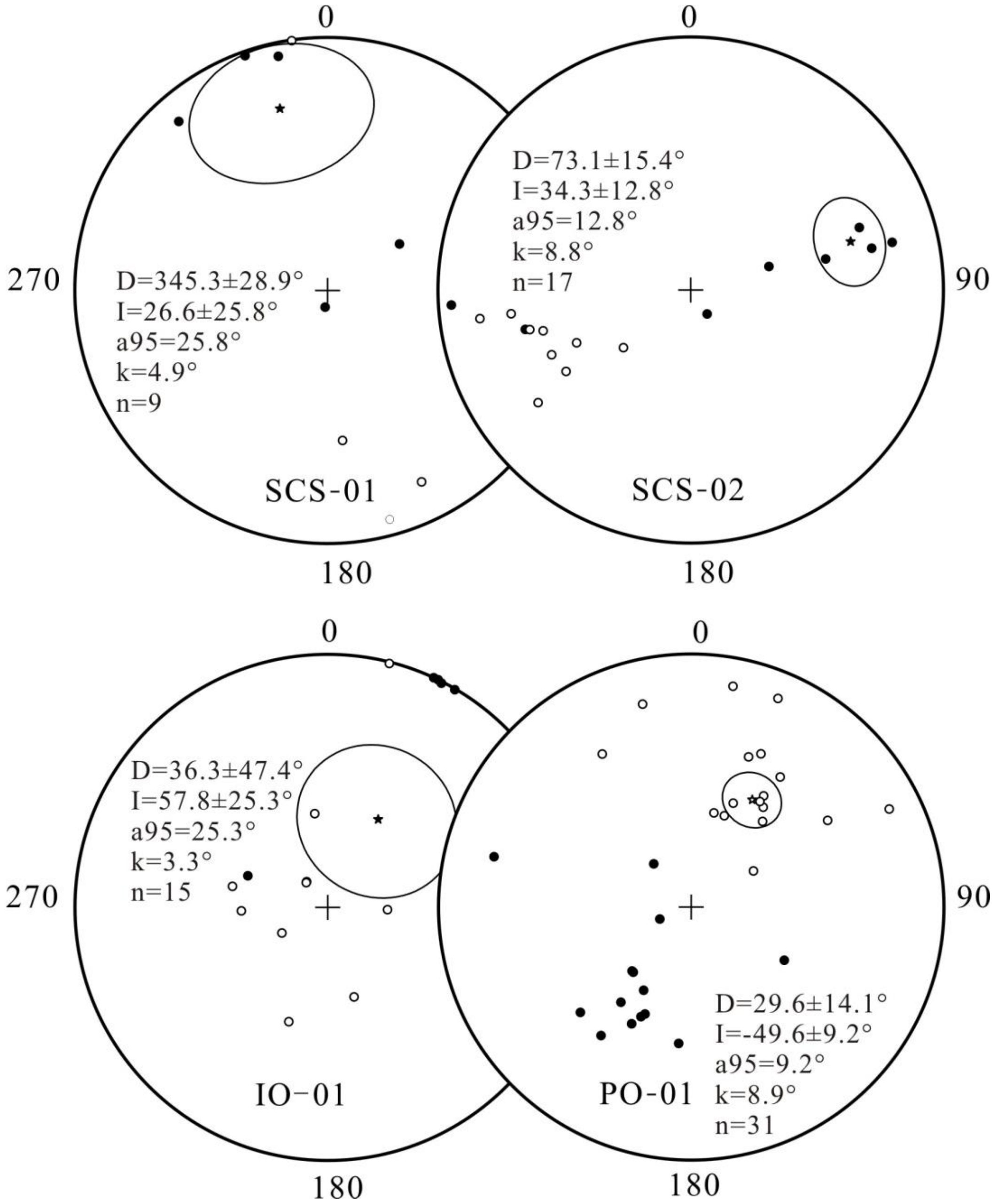

Figure S4. Equal-area projections of sample-mean directions for the characteristic remanent magnetization (ChRM) directions.

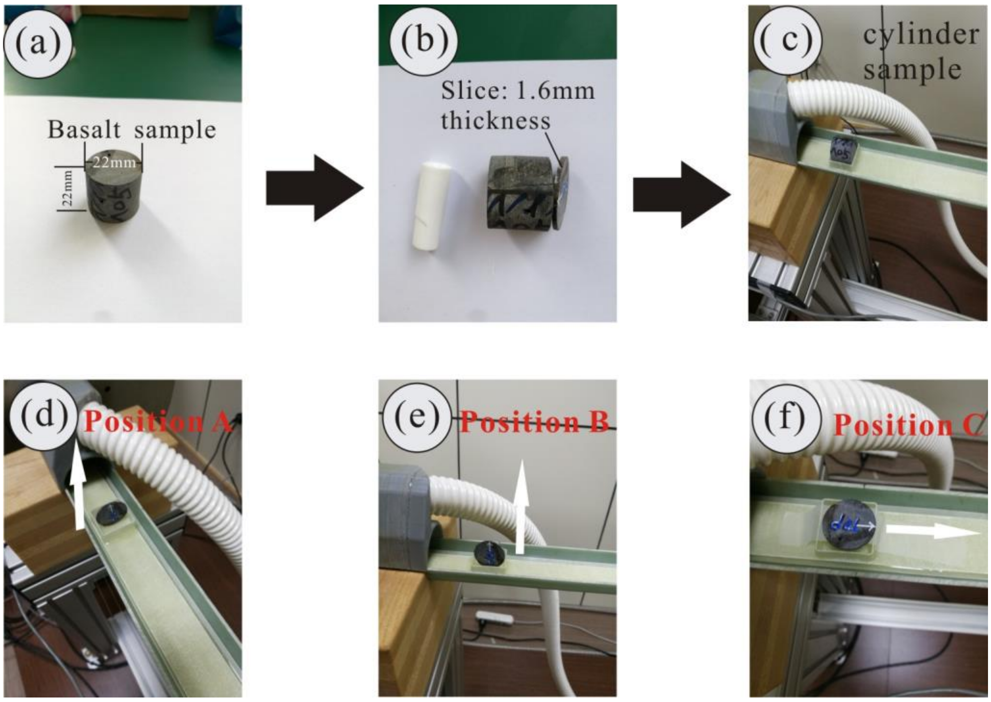

Figure S5. The NRM measurement for basalt sample of cylinder sample(c) and disk sample(d-f). The position A (d), position B (e) and position C (f) represent the flat surface of disk sample facing z, y, and x directions respectively. The position A is the same as that in the measurements for Fe-Mn crust samples.

Table S1 Summary of sampling data and the AF demagnetization results of the Fe-Mn crust samples.
* The slice samples of substrate rock; k, α95: precision parameters and half-angle of cone of 95 per cent confidence about the mean direction; s: standard deviations; n: number of samples used in statistic analysis.

Sample	Depth (mm)	Slice				Paleomagnetic Results				
		Thickness(mm)	Length(mm)	Width(mm)	Weight(g)	NRM(A/m)	Range(mT)	Declination (°)	Inclination (°)	α95 (°)
SCS-01, South China Sea (15°17′N, 117°34′E, water depth 3273 m)										
SCS-01-1	0-2.2	2.2	10.5	10.5	0.32	1.84E-03	25-50	340.6	1.9	1.1
SCS-01-2	2.62-3.62	1	10.5	10.5	0.2	6.47E-04	NRM-50	348.1	6.1	13.4
SCS-01-3	4.04-5.04	1	10.5	10.5	0.19	5.72E-03	15-30	188.1	84.6	8.5
SCS-01-4	5.46-6.56	1.1	10.5	10.5	0.17	1.84E-03	12.5-50	153.8	-16.9	15
SCS-01-5	6.98-8.18	1.2	10.5	10.5	0.18	1.37E-03	12.5-50	166.8	-8.6	17.6
SCS-01-6	8.6-9.6	1	10.5	10.5	0.12	1.62E-03	7.5-17.5	57.2	62.3	17.2
SCS-01-7	10.02-11.02	1	10.5	10.5	0.2	5.59E-04	NRM-50	154.3	-3.3	2.9
SCS-01-8	11.44-12.44	1	10.5	10.5	0.13	2.77E-04	NRM-50	174.3	-40.2	23.3
SCS-01-9	12.86-13.86	1	10.5	10.5	0.1	1.29E-04	15-30	331.1	13.1	10
normal polarity, n=6, k=3.3°, s=46.0°								346±49.9	29.6±43.4	43.4
reversed polarity, n=3, k=18.6°, s=18.9°								164.3±31.8	-22.0±29.5	29.5
average of specimens, n=9, k=4.9°, s=37.9°								345.3±28.9	26.6±25.8	25.8
SCS-02, South China Sea (15°09′N, 117°23′E, water depth 2430 m)										
SCS-02-1	0-1.08	1.08	11.04	16.29	0.099	2.48E-04	NRM-80	77	27.4	11.7
SCS-02-2	1.28-1.66	0.38	11.04	16.29	0.061	2.46E-04	10-80	69.7	29.6	11.4
SCS-02-3	1.86-2.45	0.59	11.04	16.29	0.1	2.45E-04	25-40	77.1	44.4	6.7
SCS-02-4	2.65-3.19	0.54	11.04	16.29	0.062	3.37E-04	15-35	229.5	-61.3	20.7

SCS-02-5	3.39-3.91	0.52	11.04	16.29	0.073	1.84E-04	15-50	245.2	-48.8	23.2
SCS-02-6	4.11-4.6	0.49	11.04	16.29	0.074	1.42E-04	NRM-35	262.5	-29.1	29.5
SCS-02-7	4.8-5.28	0.48	11.04	16.29	0.074	3.42E-04	15-80	256.2	-34.8	21.9
SCS-02-8	5.48-5.97	0.49	11.04	16.29	0.075	1.52E-04	20-80	254.6	-39.3	7.6
SCS-02-9	6.17-6.72	0.55	11.04	16.29	0.078	2.15E-04	10-50	233.6	-25.9	29.3
SCS-02-10	6.92-7.31	0.39	11.04	16.29	0.073	2.49E-04	15-60	245.1	-39.2	10.4
SCS-02-11	7.51-7.89	0.38	11.04	16.29	0.064	8.96E-05	20-80	73.2	63.6	20.2
SCS-02-12	8.09-8.29	0.2	11.04	16.29	0.035	5.77E-05	10-80	145.3	80.7	29.2
SCS-02-13	8.49-9.19	0.7	11.04	16.29	0.13	2.31E-04	15-50	76.7	19.2	16.6
SCS-02-14	9.39-10.24	0.85	11.04	16.29	0.15	5.92E-05	10-50	236.9	-40.8	10.8
SCS-02-15	10.44-11.36	0.92	11.04	16.29	0.19	2.79E-04	15-50	262.3	-17	8.2
SCS-02-16	11.56-12.01	0.45	11.04	16.29	0.096	1.60E-04	10-35	256.6	33.2	14.6
SCS-02-17*	12.21-14.84	2.63	11.04	16.29	0.396	1.36E-03	20-60	266.4	5.7	19.4
normal polarity, n=6, k=10.0°, s=26.0°								77.1±31.3	44.7±22.3	22.3
reversed polarity, n=11, k=8.6°, s=28.5°								251.4±18.8	-28.5±16.6	16.6
average of specimens, n=17, k=8.8°, s=28.3°								73.1±15.4	34.3±12.8	12.8
PO-01, Pacific Ocean (20.3°N, 174.2°E, water depth 2355 m)										
PO-01-1	0-2.52	2.52	14.5	15.5	0.68	4.54E-03	10-40	219.6	61.6	0.9
PO-01-2	2.94-3.94	1	14.5	15.5	0.15	2.36E-04	25-60	59.8	-66.8	2.6
PO-01-3	4.36-5.48	1.12	14.5	15.5	0.58	1.16E-03	10-60	38.9	-54.1	1.2
PO-01-4	5.90-7.00	1.1	14.5	15.5	0.52	9.74E-04	17.5-60	36.4	-38.4	8.9
PO-01-5	7.42-8.42	1	14.5	15.5	0.39	6.95E-04	20-60	216.5	51.4	4.2
PO-01-6	8.84-9.94	1.1	14.5	15.5	0.32	2.21E-04	25-50	33.1	-46.5	4.5
PO-01-7	10.36-11.46	1.1	14.5	15.5	0.44	2.37E-03	30-60	22	-53.5	2.3

PO-01-8	11.88-12.88	1	14.5	15.5	0.36	9.08E-04	25-60	19.6	-69	5.5
PO-01-9	13.30-14.30	1	14.5	15.5	0.36	5.18E-04	25-60	29.3	-46.3	24
PO-01-10	14.72-16.27	1.55	14.5	15.5	0.55	6.15E-04	NRM-20	248.8	79.6	2.1
PO-01-11	16.69-17.69	1	14.5	15.5	0.4	4.20E-04	NRM-50	224.4	61.8	3.2
PO-01-12	18.11-19.21	1.1	14.5	15.5	0.38	5.38E-04	15-50	210.5	58.7	2.6
PO-01-13	19.63-20.73	1.1	14.5	15.5	0.42	2.28E-04	30-50	63.7	-13.7	0
PO-01-14	21.15-22.25	1.1	14.5	15.5	0.34	1.47E-04	17.5-50	16.5	-12	22.7
PO-01-15	22.67-23.67	1	14.5	15.5	0.29	5.65E-04	20-50	213.5	53.2	3.9
PO-01-16	24.09-25.09	1	14.5	15.5	0.37	1.76E-04	15-50	24.5	-33.9	5.5
PO-01-17	25.51-26.51	1	14.5	15.5	0.34	9.48E-04	30-60	22	-38.9	15.2
PO-01-18	26.93-27.93	1	14.5	15.5	0.58	1.24E-04	20-50	196	49.7	13.2
PO-01-19	28.35-29.35	1	14.5	15.5	0.28	4.94E-04	30-60	194.5	48.4	9.4
PO-01-20	29.77-30.77	1	14.5	15.5	0.28	1.12E-03	30-50	13.6	-58.6	27.5
PO-01-21	31.19-32.09	0.9	14.5	15.5	0.26	3.54E-04	45-60	284.3	20.6	10
PO-01-22	32.51-33.41	0.9	14.5	15.5	0.25	9.47E-05	12.5-20	39.7	-9.9	14.6
PO-01-23	33.83-37.33	3.5	14.5	15.5	0.61	9.13E-04	30-60	125.3	50.7	10.8
PO-01-24	37.73-38.65	0.9	14.5	15.5	0.18	1.19E-04	7.5-60	215.1	38	11.8
PO-01-25	39.07-40.47	1.4	14.5	15.5	0.39	4.12E-04	15-35	201	47.1	3.5
PO-01-26	40.89-42.49	1.6	14.5	15.5	0.41	5.01E-03	NRM-60	318.9	71.6	0.6
PO-01-27	42.91-44.61	1.7	14.5	15.5	0.38	1.02E-03	30-50	120.8	-76.3	0.9
PO-01-28	45.03-46.63	1.6	14.5	15.5	0.2	5.40E-04	25-45	15.7	-13.3	8.5
PO-01-29	47.05-49.05	2	14.5	15.5	0.32	9.50E-04	25-60	38.3	-48.8	8.3
PO-01-30	49.47-51.47	2	14.5	15.5	0.3	1.66E-03	25-60	20.9	-36.6	4.8
PO-01-31	51.89-53.89	2	14.5	15.5	0.27	1.92E-04	15-40	208.5	38.4	19

normal polarity, n=14, k=9.1°, s=27.7°								217.5±27.0	58.8±14.0	14.0
reversed polarity, n=17, k=10.3°, s=25.7°								25.2±15.7	-41.9±11.7	11.7
average of specimens, n=31, k=8.9°, s=27.9°								29.6±14.1	-49.6±9.2	9.2
SWIR-S018, Indian Ocean (37°47′S, 49°45′E, water depth 2576 m)										
IO-01-1	0-0.80	0.8	14	12	0.136	1.05E-04	60-90	163.6	-59.8	10
IO-01-2	1.00-1.90	0.9	14	12	0.087	3.42E-05	50-80	92.1	-70.7	10
IO-01-3	2.10-2.88	0.78	14	12	0.23	3.16E-05	25-65	351.3	-72.9	4.5
IO-01-4	3.08-3.71	0.63	14	12	0.147	3.86E-06	50-80	320.7	-79.3	7.6
IO-01-5	3.91-4.31	0.4	14	12	0.131	4.51E-06	25-80	318.9	-79.5	7.5
IO-01-6	4.51-5.13	0.62	14	12	0.175	2.15E-05	20-80	240.8	-73.1	8.8
IO-01-7	5.33-6.09	0.76	14	12	0.164	6.93E-06	20-65	282.5	-58.3	21.4
IO-01-8	6.29-6.92	0.63	14	12	0.141	1.20E-05	20-80	198.8	-50.6	29.8
IO-01-9	7.12-7.72	0.6	14	12	0.145	1.33E-05	25-80	267.7	-62	9.7
IO-01-10	7.92-8.61	0.69	14	12	0.199	3.19E-05	40-80	352.1	-59.3	25.9
IO-01-11	8.81-9.45	0.64	14	12	0.15	3.56E-04	20-80	30.3	0.4	13.7
IO-01-12	9.65-10.14	0.49	14	12	0.151	5.02E-04	NRM-80	26.9	0.7	5.1
IO-01-13	10.34-11.35	1.01	14	12	0.298	1.96E-03	30-80	14.2	-0.7	11.2
IO-01-14*	11.55-13.55	2	14	12	1.588	1.41E-02	10-80	25.9	0	2.2
IO-01-15*	13.75-15.75	2	14	12	1.486	1.75E-02	5-80	24.8	0	3.9
normal polarity, n=10, k=10.6°, s=25.2°								267.2.0±180.0	-81.7±15.6	15.6
reversed polarity, n=5, k=177.1°, s=6.1°								24.4±5.8	0.1±5.8	5.8
average of specimens, n=15, k=3.3°, s=46.5°								36.3±47.4	57.8±25.3	25.3

Table S2 Summary of NRM data and directional difference analysis between cylinder sample and disk samples. The position A, B and C represent the flat surface of disk sample facing z, y, and x directions respectively (Figure S5). The position A is the same as that in the measurements for Fe-Mn crust samples.

Sample/Orientation Information	Sample	17m054	17m054		17m054a	17m054a		17m054b	17m054b		17m054c	17m054c	
	Number	1	2		1	2		1	2		1	2	
	Shape	Cylinder	Cylinder		Disk	Disk		Disk	Disk		Disk	Disk	
	Position	-	-		A	A		B	B		C	C	
	Orientation	Top facing away from SRM(A/m)	Top facing away from SRM(A/m)	Average (A/m)	Top facing away from SRM(A/m)	Top facing away from SRM(A/m)	Average (A/m)	Top facing away from wall(A/m)	Top facing away from wall(A/m)	Average (A/m)	Top facing upward (A/m)	Top facing upward (A/m)	Average (A/m)
SRM corrdinate	x	-5.20E-02	-5.39E-02	-5.30E-02	-4.27E-03	-4.27E-03	-4.27E-03	-4.24E-03	-4.15E-03	-4.19E-03	7.50E-04	5.80E-04	6.65E-04
	y	-3.01E-02	-2.46E-02	-2.73E-02	-1.56E-03	-1.55E-03	-1.56E-03	-6.45E-04	-4.67E-04	-5.56E-04	9.97E-04	1.20E-03	1.10E-03
	z	-7.47E-03	-7.22E-03	-7.34E-03	-3.55E-04	-3.45E-04	-3.50E-04	1.51E-03	1.79E-03	1.65E-03	4.31E-03	4.34E-03	4.32E-03
Sample coordinate	x'	-5.20E-02	-5.39E-02	-5.30E-02	-4.27E-03	-4.27E-03	-4.27E-03	-4.24E-03	-4.15E-03	-4.19E-03	-4.31E-03	-4.34E-03	-4.32E-03
	y'	-3.01E-02	-2.46E-02	-2.73E-02	-1.56E-03	-1.55E-03	-1.56E-03	-1.51E-03	-1.79E-03	-1.65E-03	-9.97E-04	-1.20E-03	-1.10E-03
	z'	-7.47E-03	-7.22E-03	-7.34E-03	-3.55E-04	-3.45E-04	-3.50E-04	-6.45E-04	-4.67E-04	-5.56E-04	-7.50E-04	-5.80E-04	-6.65E-04
Sample coordinate	Declination (°)	210.1	204.5	207.3	200.0	200.0	200.0	199.6	203.3	201.5	193.0	195.5	194.3
	Inclination (°)	-7.1	-6.9	-7.0	-4.5	-4.3	-4.4	-8.2	-5.9	-7.0	-9.6	-7.3	-8.5
	Intensity(A/m)	6.05E-02	5.97E-02	6.01E-02	4.56E-03	4.56E-03	4.56E-03	4.54E-03	4.54E-03	4.54E-03	4.48E-03	4.54E-03	4.51E-03
Unit Vector	x	-8.59E-01	-9.03E-01	-8.82E-01	-9.37E-01	-9.37E-01	-9.37E-01	-9.32E-01	-9.13E-01	-9.23E-01	-9.61E-01	-9.56E-01	-9.59E-01
	y	-4.97E-01	-4.12E-01	-4.55E-01	-3.41E-01	-3.41E-01	-3.41E-01	-3.33E-01	-3.94E-01	-3.64E-01	-2.22E-01	-2.65E-01	-2.44E-01
	z	-1.23E-01	-1.21E-01	-1.22E-01	-7.78E-02	-7.56E-02	-7.67E-02	-1.42E-01	-1.03E-01	-1.23E-01	-1.67E-01	-1.28E-01	-1.47E-01
Difference from cylinder sample	Inner Product						0.991			0.995			0.974
	Angle (°)						7.7			5.8			13.0

Difference from A Position	Inner Product									0.999			0.993
	Angle (°)									3.0			7.0
Difference from B Position	Inner Product												0.992
	Angle (°)												7.3
